# Editorial: Oral health and care in the elderly population and aging society

**DOI:** 10.3389/fdmed.2025.1745526

**Published:** 2025-12-29

**Authors:** Sherry Shiqian Gao, Melissa Adiatman

**Affiliations:** 1 Department of Stomatology, School of Medicine, Xiamen University, Xiamen, China; 2 Faculty of Dentistry, University of Indonesia, Jakarta, Indonesia

**Keywords:** ageing, oral health, elderly, quality of life, disparities

In the past decades, because improvements in living quality have contributed to a significant increase in life expectancy, the ageing population has become an important issue all over the world (1). Oral health problems, including dental caries and periodontal diseases, are prevalent in the elderly population. Both untreated dental caries and periodontal diseases will affect the whole dentition, leading to compromised masticatory function; poor chewing ability and discomfort in eating will affect the absorption of nutrients and cause long-term malnutrition (2). Meanwhile, missing teeth or edentulous will influence the appearance, self-esteem, interpersonal communication of elderly patients and quality of life (3). In addition, periodontitis is a risk factor for some systemic conditions, including Alzheimer's disease, upper respiratory infections, pneumonia, etc. (4). It is noteworthy that poor oral health may also compromise the general health of middle-aged people and result in biological ageing in this group, even though they are not counted as the elderly population (5). Therefore, the high prevalence of oral health problems will cause a significant disease burden to the health system. To address the oral health problems in the elderly population and ageing society, dentists, dental researchers and dental public health sectors should understand the oral health situations of elderly people, assess their oral health care needs and develop effective oral health care strategies.

This Research Topic has collected five outstanding publications from China, Japan, Sweden, and the United States, which gather worldwide perspectives regarding oral health issues of the elderly population. Chen et al. performed a cross-sectional study to investigate the association between oral health status and frailty in Chinese older adults. This study adopted a chain mediating model and identified sleep quality and depressive symptoms as the two mediators that associated with the relationship between oral health status and frailty in older adults. The results suggested that potential interventions should be targeted in oral health care, sleep improvement and mental health management, so that to assist in slowing the frailty process. Another Chinese study focused on a more specific group of elderly people with dental implants. Huang et al. conducted a randomised trial to investigate the effectiveness of experiential education combined with health coaching techniques (EE + HCT) in improving oral health in elderly patients with dental implants. Although there was no difference regarding implant survival rates between the EE + HCT and the control groups, EE + HCT could enhance oral health self-efficacy, oral hygiene practices, and peri-implant periodontal condition compared to the control group. This study demonstrated that EE + HCT can be a promising strategy to improve periodontal health in elderly patients after dental implant treatment. Lowenstein et al. provided a mini review discussing the disparities in oral health access and outcomes for elderly adults in the United States. This study focused on three domains: 1) assessing demographic disparities in access to oral health services, 2) analysing the relationship between oral literacy and preventive care behaviours and health outcomes, and 3) evaluating the consequences of oral diseases, such as nutrition intake and broader health outcomes. Based on the results, the authors proposed recommendations, including health literacy improvements, community-based programs and policy and insurance reforms, to reduce disparities and improve oral health outcomes for aging adults. Kiswanjaya et al. conducted a cross-sectional study investigating the relationship between age and mandibular morphological changes in adults and elderly adults in Japan. The results showed that adults aged 65 or older presented increased cortical erosion and decreased cortical width, and age was an important predictor of mandibular cortical index. This study demonstrated that older individuals had greater cortical degradation; specific approaches should be considered for this high-risk group of people. Neves-Guimaraes et al. used a cohort profile to investigate the validity of self-reported health data from elderly individuals in Sweden. This study matched the self-reported data with register-based data, evaluated the agreement between them, and identified factors associated with non-response and drop-out cases. The results showed that elderly being male and an immigrant, having a lower income and education level, being single, and presenting poor health were predictors of non-response and drop-out cases.

In conclusion, this Research Topic identified studies with good qualities investigating oral health-related issues in elderly people. Dental professionals and clinicians can refer to those studies to obtain up-to-date information regarding elderly oral health care.
